# From evidence to action: Gender-sensitive cardiovascular care – A quantitative survey with physicians working in cardiology departments

**DOI:** 10.1371/journal.pgph.0006357

**Published:** 2026-04-29

**Authors:** Sophia Sgraja, Judith Mollenhauer, Ute Seeland, Martina Kloepfer, Clarissa Kurscheid, Volker E. Amelung

**Affiliations:** 1 Institute for Epidemiology, Social Medicine and Health Systems Research, Hannover Medical School, Hanover, Germany; 2 figus – Research Institute for Health- and System Design, Cologne, Germany; 3 Medical Faculty University Hospital Magdeburg Otto von Guericke, Magdeburg, Germany; 4 Institute for Gender Health, Berlin, Germany; King’s College London, UNITED KINGDOM OF GREAT BRITAIN AND NORTHERN IRELAND

## Abstract

Gender-sensitive care (GSC+) is a key component of personalized medicine and essential for ensuring quality and equity in cardiovascular care. By integrating biological, social, and cultural dimensions of gender as well as diversity-related factors, GSC + can enhance diagnostic accuracy and therapeutic effectiveness. Although cardiovascular guidelines increasingly incorporate sex- and gender-specific recommendations, little is known about physicians’ awareness, knowledge, and the extent to which GSC + is implemented in practice. This study aimed to assess physicians’ gender sensitivity, knowledge of sex- and gender-specific guideline content, and the perceived implementation of GSC+ in inpatient cardiology settings. A cross-sectional online survey was conducted among physicians working in German inpatient cardiology wards (n = 155). The questionnaire assessed gender sensitivity (N-GAMS), knowledge of sex- and gender-specific cardiovascular guideline content, and perceived implementation of in clinical practice. Descriptive, correlational, and regression analyses were performed. Physicians demonstrated high gender sensitivity (M = 3.95, SD = 0.73) and moderate-to-high knowledge of guideline content (M = 0.63, SD = 0.08). Specific knowledge gaps were identified. The perceived implementation of GSC+ in clinical practice was rated as moderate to low (M = 2.45, SD = 0.59). Higher knowledge levels were observed among physicians working in university hospitals, while differences in gender sensitivity were observed with respect to country of birth. Importantly, no significant relationships were found between gender sensitivity, knowledge, and perceived implementation, indicating a disconnect between awareness, knowledge, and clinical application. The findings indicate a gap between existing scientific evidence and its translation into practice. While physicians exhibit high awareness and adequate knowledge, this does not consistently translate into implementation. This discrepancy underscores structural and systemic barriers to integrating GSC+ into care. Strengthening the systematic incorporation into clinical guidelines, medical education, and institutional quality frameworks may enhance implementation, reduce diagnostic and therapeutic disparities, and promote more equitable, evidence-based cardiovascular care.

## 1. Introduction

The consideration of sex and gender in healthcare is a key component of personalized, evidence-based medical practice. Gender-sensitive care (GSC+) addresses not only biological and physiological differences between the sexes, but also the social and cultural dimensions of gender. Social dimensions include, e.g., gender roles, working and living conditions, and health-related behaviors. Cultural dimensions involve norms, communication patterns, and attitudes towards illness and care. The “+” in GSC+ stands for additional diversity-related factors such as age, ethnicity, socioeconomic status, physical and mental health, religion, and sexual identity [1]. For physicians, awareness of these intersecting factors is essential to provide equitable, individualized care, improve patient outcomes, and reduce systemic disparities. In this sense, GSC+ implies moving beyond ‘one-size-fits-all’ approaches towards more stratified, target-group–specific approaches to healthcare. These approaches better reflect the heterogeneity of patient populations and strengthen the precision and effectiveness of medical care.

Despite increasing recognition of the importance of sex, gender, and intersectionality in medicine, little is known about how healthcare providers understand these concepts, are aware of their importance, or incorporate them into their daily clinical practice. This potential discrepancy between theoretical frameworks and practical implementation underscores the need for a systematic assessment of gender-related knowledge and awareness among healthcare providers to address an existing research gap.

Cardiovascular diseases remain one of the leading causes of death worldwide. The field of cardiology was the first medical discipline to systematically explore sex-based differences in disease patterns, clinical presentations, and treatment responses. It was catalyzed by the pioneering work of researchers such as Marianne J. Legato in the US in the 1990s [2]. Since then, a growing body of research has documented substantial sex- and gender-related disparities across the entire continuum of cardiovascular care, including symptom recognition, diagnostic accuracy, pharmacological efficacy, and treatment outcomes [3]. These disparities can translate directly into delayed diagnoses, suboptimal treatment decisions, and preventable differences in morbidity and mortality for women and gender-diverse individuals. Insufficient attention to sex- and gender-specific differences, therefore, can have particularly severe consequences for healthcare outcomes. Research further shows that gender influences early clinical decision-making, including the legitimacy and urgency attributed to patient-reported symptoms such as pain [7]. Another aspect is that symptom communication can vary by gender, which may affect how clinical information is interpreted during early patient encounters. Differences in how symptoms are articulated may not always align with dominant biomedical expectations of concise reporting, potentially influencing initial diagnostic judgments. These examples highlight that gender-related disparities arise at multiple points along the clinical pathway, not only in treatment decisions but also in the earliest stages of patient assessment.

The growing evidence of sex- and gender-specific differences has begun to be reflected in clinical guidelines of cardiology (A consolidated summary of sex- and gender-specific recommendations from German and European cardiology guidelines relevant to myocardial infarction is provided in Appendix 2 of this document.). The extent and specificity of sex- and gender-sensitive recommendations vary considerably across countries and professional societies. Clinical practice guidelines represent a key source of such knowledge and form the basis for appropriate, gender-sensitive care decisions [4,5].

A comprehensive understanding of sex- and gender-related differences in medicine requires not only factual knowledge, but also professional awareness and acceptance of their relevance. Gender awareness among healthcare professionals can be assessed using the Nijmegen Gender Awareness in Medicine Scale (N-GAMS), which captures three dimensions: gender sensitivity, gender role ideology toward patients, and gender role ideology toward healthcare professionals. Originally developed for medical students, the N-GAMS has since been validated across various healthcare professions and cultural contexts, demonstrating strong psychometric robustness and confirming its suitability as a reliable instrument for measuring gender awareness in clinical settings [6–9]. Existing research suggests that gender awareness is shaped by cultural, educational, and institutional factors, resulting in a substantial variability across healthcare professional groups and countries [6–9].

The study from Steinböck among medical students illustrates that even when gender awareness is present overall, gender-based stereotypes, particularly toward patients, persist. Such enduring stereotypes may influence future diagnostic reasoning and therapeutic decisions, underscoring that gender awareness does not automatically translate into stereotype-free clinical practice [9]. These findings highlight the importance of empirically assessing gender awareness not only among trainees but also among fully practicing clinicians, whose decision-making directly impacts patient outcomes. Despite its international relevance, N-GAMS has rarely been used to assess gender awareness specifically among physicians in cardiology, a group whose awareness of sex- and gender-specific factors is critically important for accurate diagnosis and treatment in a specialty characterized by well-documented gender disparities. This gap reinforces the need for systematic assessment of gender awareness in this specific clinical population. At the same time, the relevance of such assessments is amplified by broader policy developments that increasingly frame gender sensitivity as a core component of high-quality care.

This dimension of gender awareness is particularly important in light of international and national health policy agendas such as the World Health Organization’s strategy for gender mainstreaming in health systems, which increasingly call for the systematic integration of sex and gender considerations in healthcare education, service delivery, and policy development [10–12].

The health services research project *HeartGap* (Gender Health Gaps in guideline-based inpatient cardiovascular care and implementation strategies to reduce these gaps), funded by the Innovation Fund of the Federal Joint Committee (grant number 01VSF22030), systematically investigates the implementation of GSC+ in cardiology wards in German hospitals. As an initial exploratory study, it aims to

(1) assess physicians’ gender sensitivity,
(2) evaluate their knowledge of sex- and gender-specific cardiovascular aspects,
(3) examine their perceptions of the implementation of gender-sensitive healthcare (GSC+) in clinical practice, and
(4) identify barriers and facilitators to its integration into clinical care [13].

By examining these dimensions, the study addresses a critical research gap and provides insights into knowledge deficits and structural or attitudinal barriers to the implementation of gender-sensitive cardiovascular care. Its findings provide urgently needed insights into where gender-sensitive care is successfully practiced, where knowledge deficits persist, and which structural or attitudinal barriers hinder the integration of GSC+ into clinical cardiology practice. These results can inform the development of targeted educational measures, guide the design of implementation strategies, and ultimately improve guideline-based cardiovascular care for diverse patient populations.

The process of systematically extracting, translating, and consolidating sex- and gender-specific content from multiple cardiology guidelines, undertaken as part of the knowledge assessment in this study, may itself offer added value for physicians. By providing a concise and accessible summary of dispersed guideline recommendations, this material can help make relevant content more visible and easier to integrate into routine clinical decision-making. A structured summary of these guideline elements is available in S1_Text to support transparency and practical usability.

## 2. Methods

### 2.1 Ethics statement

Ethical approval for this study was obtained from the Ethics Committee of Hannover Medical School (Approval No. 10745_BO_K_2023). All procedures were conducted in accordance with the Declaration of Helsinki and relevant national data protection regulations. Participation was voluntary, and informed consent was obtained electronically from all respondents prior to the start of the survey.

### 2.2 Study design and hypotheses

To explore how GSC+ is currently understood and applied in cardiology, a cross-sectional, quantitative survey was conducted among physicians working in cardiology wards. The study aimed to assess physicians’ gender awareness, their knowledge of sex- and gender-specific aspects of cardiovascular medicine, and their perceptions of how GSC + is implemented in everyday clinical practice.

**Fig 1:**
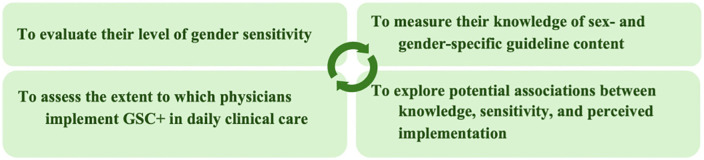
Study objectives related to gender sensitivity, knowledge, and implementation of GSC+.

**Fig 2:**
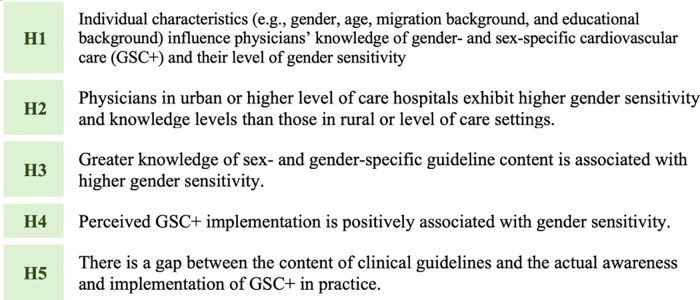
Study hypotheses (H1–H5) on gender sensitivity, knowledge, and implementation of GSC + .

The study objectives are summarized in Fig 1:

The hypotheses tested in this study are summarized in Fig 2:

### 2.3 Sample and data collection

Data were collected between May 2024 and February 2025 using an anonymous online questionnaire, which was administered on the Platform LimeSurvey. The survey targeted physicians working in cardiology and was distributed via a recruitment strategy. Hospitals with cardiology departments or internal medicine units focusing on cardiology were identified through the German national hospital directory.

In Germany, hospitals are typically classified by the level of care they provide. University hospitals and large tertiary care hospitals (>800 beds) provide the highest level of specialized and highly differentiated services, often including research and teaching responsibilities. Secondary care hospitals (501–800 beds) deliver a broad spectrum of services but with fewer subspecialties, while primary care hospitals (<500 beds) generally cover basic and common medical needs, with limited specialization. In addition, the regional context, urban, town, or rural, influences both the availability of medical resources and access to specialized cardiovascular care [14].

Physicians were recruited through direct email invitations to hospitals, professional medical networks and associations, and newsletters. Eligible participants were physicians engaged in active clinical cardiology practice, employed at a hospital in Germany, and willing to participate voluntarily. Written study information was provided at the beginning of the survey, and participants confirmed informed consent before proceeding.

To ensure sample representativeness and allow for post-stratification weighting, hospitals and participants were categorized by level of care (university with >800 beds, tertiary >800 beds, secondary with 501–800 beds, or primary care <500 beds) and by regional location (urban ≥100,000 inhabitants, town <100,000, or rural <5,000). Hospital characteristics used for stratification are shown in Table 1. These strata were later used to calculate sample weights.

**Table 1:** Attributes of included hospitals [13].

		*Level of care*			
***Population size***		**Teaching/ research hospital** (> 800 beds)	**Hospital** (> 800 beds)	**Hospital** (501 – 800 beds)	**Hospital** (≤ 500 beds)
	**Large city** (≥ 100,000 inhabitants)				
	**City** (5,000–99,000 inhabitants)				
	**Rural community** (< 5,000 inhabitants)				

### 2.4 Survey instrument

The digital questionnaire was divided into three sections: Sociodemographics, gender sensitivity and knowledge from the medical guidelines. The translated version of the questionnaire is available in S1 Questionnaire.

The first section collected background data on respondents’ gender, age, migration background, highest educational degree, medical qualification, and years of clinical experience in cardiology. It also captured structural context variables (see Table 1). These variables were later used to stratify the sample and explore subgroup differences.

The second part assessed physicians’ gender sensitivity using the 13-item gender sensitivity (GS) subscale from the Nijmegen Gender Awareness in Medicine Scale (N-GAMS). The N-GAMS Scale was originally designed for medical students and later adapted and validated for physicians. Items were rated on a 5-point Likert scale (1 = strongly disagree to 5 = strongly agree). Negatively worded items were reverse-coded. A higher mean score indicates greater gender sensitivity.

The final section of the questionnaire consisted of a factual knowledge test. A total of 17 self-developed binary-coded items (true/false or multiple-choice) assessed knowledge of sex- and gender-specific aspects of cardiovascular care. Item development was based on a systematic screening of established clinical guidelines relevant to myocardial infarction and acute coronary syndromes, including those from the European Society of Cardiology (ESC), the German Cardiac Society (DGK), and the Association of the Scientific Medical Societies in Germany (AWMF). An overview of the clinical guidelines used to develop the knowledge items is provided in Table 2.

**Table 2:** Medical Guidelines used for Screening (translated from german).

German Cardiac Society (DGK) Guidelines:
Therapy of acute myocardial infarction in patients with ST-segment elevation (STEMI)
Non–ST-segment elevation acute coronary syndrome (NSTE-ACS)
Guidelines of the Association of the Scientific Medical Societies in Germany (AWMF):
S3 Guideline – National Disease Management Guideline for Chronic Coronary Heart Disease (Chronic CHD).
European Society of Cardiology (ESC) Guidelines:
(2020) Acute Coronary Syndromes (ACS) in Patients Presenting without Persistent ST-Segment Elevation – European Society of Cardiology Guidelines
(2023) ESC Guidelines for the Management of Acute Coronary Syndromes – Developed by the Task Force for the Management of Acute Coronary Syndromes of the European Society of Cardiology (ESC)

To identify relevant sex- and gender-specific content, the guidelines were systematically screened using targeted keyword searches for *sex*, *gender*, *man*, *men*, *woman*, and *women*. All identified passages describing sex- or gender-related differences in clinical presentation, diagnostics, therapy, outcomes, or risk factors were systematically documented. Across AWMF, DGK and ESC cardiology guidelines, sex- and gender-related differences were identified in several clinically relevant domains. These included differences in disease prevalence (e.g., higher lifetime prevalence of CHD in men), symptom presentation (more atypical or non-chest-pain symptoms in women), diagnostic accuracy (lower specificity of exercise ECG in women), biomarker interpretation (sex-specific hs-cTn thresholds), and pathophysiology (e.g., higher prevalence of MINOCA in women). Further guideline content highlighted sex-dependent treatment considerations, such as differential protective effects of statin therapy in primary prevention, sex-associated bleeding risks in antithrombotic treatment, and the influence of sex and age in risk stratification tools (e.g., CHA₂DS₂-VASc). Additional aspects included sex-specific thresholds for ST-segment elevation, diagnostic nuances in acute coronary syndromes, pregnancy-related cardiovascular risks, and special considerations for the correct placement of ECG electrodes in women.

The instrument was designed to assess guideline-based factual knowledge rather than to constitute a psychometrically validated scale. Based on this material, knowledge items were developed in collaboration with a specialist in gender medicine and subsequently refined through expert review to ensure clinical relevance, clarity, and appropriateness. In addition, pilot testing was conducted with five cardiology physicians to assess comprehensibility, clarity, and feasibility of the items in a clinical context.

This content-driven and expert-informed approach supports both content and face validity, as all items directly reflect clinically relevant, guideline-derived knowledge and were systematically selected to represent key domains of sex- and gender-specific cardiovascular care, including diagnostics, treatment, and clinical presentation. The inclusion of expert review and pilot testing further ensured clinical relevance, clarity, and interpretability for the target population.

To ensure transparency and replicability of the instrument design, the complete questionnaire, as well as the extracted and consolidated guideline content on which the knowledge items are based, are provided in the appendix. This makes it possible to trace the development of all survey components and understand precisely how the guideline-derived knowledge items were constructed.

Participant responses were coded dichotomously and aggregated into a standardized knowledge index ranging from 0 (no correct responses) to 1 (all correct responses).

Perceived implementation of gender- and diversity-sensitive care was measured using a single Likert-type item. Respondents rated the extent to which gender- and diversity-sensitive care was implemented on their ward, including aspects such as respect for patients’ privacy, consideration of religious needs, and accommodation of migration-related factors (e.g., language barriers or dietary practices).

This item was designed to capture an initial, global impression of perceived implementation of gender- and diversity-sensitive care at the ward level. The aim was not to provide a comprehensive measurement of implementation, but rather to assess respondents’ subjective perception, which may vary depending on individual interpretation and experience. Given this exploratory and comparative objective, a single-item measure was considered appropriate to capture a general perception while maintaining feasibility within a multi-component survey.

Two optional open-ended questions complemented this section of the questionnaire, allowing participants to elaborate on their views regarding the role of gender in physician–patient communication and additional comments about the topic.

### 2.5 Data preparation and weighting

Following the completion of data collection, responses from the online survey (LimeSurvey) were reviewed and processed to ensure completeness and data quality. The stratification variables and post-stratification weighting approach used in the analysis are shown in Table 3. Only questionnaires in which participants had completed at least the sociodemographic and N-GAMS sections were included in the analysis. Surveys with excessive missing values were excluded. In total, n = 165 questionnaires were submitted, of which n = 155 valid cases were retained for analysis after data cleaning.

**Table 3:** Strata and weighting of data.

Strata	A priori Calculation ^a^	Sample ^a^	Difference ^b^	Weight
University hospital *- metropolitan area*	12.30	21.29	-8.99	0.58
University hospital *- city*	2.36	7.10	-4.74	0.33
Tertiary care *- metropolitan area*	2.88	10.32	-7.44	0.28
Tertiary care *- city*	0.79	0.65	0.14	1.22
Secondary care *- metropolitan area*	8.64	27.10	-18.46	0.32
Secondary care *- city*	4.97	5.81	-0.83	0.86
Primary care *- metropolitan area*	20.16	18.71	1.45	1.08
Primary care *- city*	46.86	8.39	38.47	5.59
Primary care *- rural area*	1.05	0.65	0.40	1.62

*Note:* ^a^ In percent. ^b^ In percent points.

Negatively worded items from the N-GAMS scale were reverse-coded, and gender sensitivity and knowledge indices, as well as the single-item measure of perceived implementation, were calculated as described in Section 2.3.

To address deviations from the intended stratified sampling design, post-stratification weighting was applied to the descriptive analyses. Strata were defined by hospital level (university, tertiary, secondary, primary) and regional location (urban ≥100,000; town <100,000; rural <5,000 inhabitants). Based on a priori assumptions (n = 400), relative frequencies were compared with those observed in the realized sample, and weight factors were calculated as the ratio of expected to actual stratum proportions. Table 3 presents the resulting stratum-specific weights.

All descriptive results are reported using weighted data. Regression analyses were conducted on unweighted data, as the stratification variables were included directly as covariates in the models.

### 2.6 Statistical analyses

All analyses were carried out using IBM SPSS Statistics (version 29). Descriptive statistics were used to summarize the characteristics of the physician sample, including absolute and relative frequencies for categorical variables and means with standard deviations for continuous variables.

Gender sensitivity was assessed using the N-GAMS subscale, which includes 13 items rated on a five-point scale. Negatively worded items were recoded before calculating a mean score for each respondent. Knowledge of sex- and gender-specific cardiovascular aspects was measured using 17 binary-coded items (true/false or multiple-choice), and the proportion of correct responses was aggregated into a standardized index ranging from 0 (no correct answers) to 1 (all correct). Perceived implementation of gender-sensitive care was assessed via a single-item rating on a four-point scale.

Because the survey was configured to require responses to all scale items, no item-level missing data were present for the N-GAMS, knowledge, or implementation measures. Spearman’s rank correlation was selected to examine bivariate associations because several key variables were measured on ordinal scales and did not meet the assumptions required for parametric correlation coefficients. Statistical significance was set at p < 0.05 (two-tailed) [15].

In line with methodological guidance, inferential analyses were performed on unweighted data, as the stratification variables were explicitly modeled as covariates in the linear regression analyses, rendering weighting not necessary [16]. Weighted data were used solely for descriptive summary statistics.

To identify possible influencing factors, two multiple linear regression models were conducted, one predicting gender sensitivity and one predicting perceived implementation. Predictors were selected based on theoretical considerations regarding potential confounders, including sociodemographic characteristics, clinical experience, and institutional context, which are commonly used in healthcare research [17]. Independent variables included age, gender, migration background, education level, years of professional experience, level of care, and regional setting. In the gender sensitivity model, the knowledge score was included as an additional predictor. Model specification followed established recommendations for regression analyses, ensuring an adequate case-to-predictor ratio (≥10–15 observations per predictor). A backward elimination procedure was used to remove non-significant variables (p > 0.10) from the initial models to obtain parsimonious and interpretable models. Final models retained all statistically or theoretically relevant predictors.

The dataset generated and analyzed during the current study is publicly available in the MHH Research Data Repository and can be accessed via the following DOI: https://doi.org/10.26068/mhhrpm/20251204-000

### 2.7 Analysis of open-ended responses

In addition to the quantitative survey items, the questionnaire included two optional open-ended questions intended to capture supplementary contextual information from participants. These items were designed to elicit (1) physicians’ perspectives on the role of gender in physician–patient communication and (2) general comments regarding gender-sensitive care. Because the questions were exploratory and embedded in the quantitative scale, the open-text data were not analyzed using a formal qualitative methodology.

All open-ended responses were reviewed descriptively to identify recurrent ideas, illustrative examples, and noteworthy viewpoints. This approach follows a qualitative descriptive logic, in which responses are summarized with minimal interpretation to enhance the contextual understanding of the quantitative findings. No coding framework was applied, and the results are therefore not presented as qualitative themes, but rather as narrative summaries supported by selected exemplar quotations. The purpose of these summaries is to provide insight into the range of perspectives expressed by participants. Participation in the open-text items was voluntary; thus, all responses stem from the same sample as the quantitative analysis, but represent a self-selected subset of respondents. As such, these data are interpreted solely as contextual and illustrative, complementing but not integrating analytically with the quantitative results.

## 3. Results

### 3.1 Sample characteristics

A total of 155 questionnaires were included in the analysis. The sample consisted of physicians working in cardiology wards, with a balanced gender distribution (46.0% female, 54.0% male) and a broad age range (26–65 years; M = 41.8, SD = 10.5). Participants reported varying levels of professional experience (M = 18.0 years, SD = 10.2), indicating a heterogeneous sample in terms of clinical seniority. The majority of respondents were born in Germany (73.4%). Most participants had a high level of formal education, with nearly all holding a university entrance qualification. A substantial proportion had completed an academic degree, often at doctoral level.

Most physicians worked in primary care hospitals, and the sample predominantly represented urban and metropolitan regions. Detailed sociodemographic and institutional characteristics of the sample are presented in Table 4.

**Table 4:** Distribution of sociodemographic characteristics of physicians.

Variable	n (%)	M	SD	Range
Gender	155 (100)			
Female	71 (46.0)			
Male	84 (54.0)			
Age in years	155 (100)	41.8	10.5	26–65
26-30	26 (16.9)			
31-40	48 (30.7)			
41-50	50 (32.4)			
51-60	22 (14.3)			
61-65	9 (5.7)			
Country of birth	155 (100)			
Germany	114 (73.4)			
Other	41 (26.6)			
School leaving certificate	155 (100)			
(Technical) university entrance qualification	154 (99.1)			
Secondary school certificate	1 (0.7)			
Other	0 (0.2) ^**a**^			
Professional qualification	154 (100)			
Postdoctoral lecture qualification (Habilitation)	8 (4.9)			
Doctorate	72 (46.8)			
(Technical) university degree	67 (43.5)			
Completed vocational training	7 (4.7)			
Years of professional experience	152 (100)	18.0	10.2	1-46
1-10	48 (31.8)			
11-20	39 (25.5)			
21-30	44 (29.2)			
31-46	20 (13.5)			
Hospital size	155 (100)			
University hospital	23 (14.7)			
Tertiary care hospital	6 (3.7)			
Secondary care hospital	21 (13.6)			
Primary care hospital	105 (68.1)			
Region size	155 (100)			
Large city	68 (44.0)			
City	85 (55.0)			
Rural community	2 (1.0)			

*Note:* The data are weighted by care level and region size.

M = mean; SD = standard deviation.

^a^Some respondents selected „Other“; due to weighting, the frequency was rounded to 0.

With respect to the type/size of the hospital, 14.7% of physicians worked in teaching hospitals (>800 beds), 3.7% in maximum-care facilities (>800 beds), 13.6% in specialized care hospitals (501–800 beds), and 68.1% in basic-care institutions (<=500 beds). In terms of geographical setting, 44.0% of the respondents worked in metropolitan areas (>100,000 inhabitants), 55.0% in urban areas (5,000–100,000 inhabitants), and 1.0% in rural communities (<5,000 inhabitants).

### 3.2 Gender awareness (N-GAMS)

The validated N-GAMS scale measures physicians’ awareness, attitudes, and behaviors related to gender sensitivity in medical contexts [18]. Table 5 presents the means and standard deviations for each item of this scale, based on responses from a sample of 155 physicians working in cardiology wards. Responses were rated on a scale from 1 (low gender sensitivity) to 5 (high gender sensitivity). The overall mean score of 3.95 (SD = 0.73) indicates a high level of gender awareness among the participants.

**Table 5:** Survey on sensitivity (N-GAMS) of physicians.

GS	Item	M	SD
1	A sound knowledge of gender differences among physicians improves the quality of medical care.	4.44	0.91
2 ^a^	Physicians should only deal with biological differences between men* and women*.	3.76	1.20
3 ^a^	In the case of non-gender-specific complaints, the patient’s gender plays no role.	3.57	1.24
4 ^a^	Physicians should primarily focus on purely medical aspects of health complaints from men* and women*.	3.80	1.08
5 ^a^	Physicians do not need to know what men* and women* experience in life in order to provide medical care.	4.10	0.98
6 ^a^	Differences between female and male physicians are too minor to be relevant.	4.06	1.01
7 ^a^	Precisely because men* and women* differ, physicians should treat all patients equally.	3.54	1.22
8 ^a^	Physicians who address gender differences do not deal with truly important topics.	4.12	1.02
9 ^a^	In communication with patients, it makes no difference to physicians whether the patient is male or female.	3.88	1.08
10 ^a^	In communication with patients, it does not matter whether the physician is male or female.	3.80	1.09
11 ^a^	Differences between male and female patients are so minor that physicians can hardly consider them.	4.18	0.92
12	Physicians should address gender differences in disease causes and consequences to ensure effective treatment.	3.91	1.07
13 ^a^	When describing complaints, it is not necessary to consider gender differences.	4.18	0.98

*Note:* Item scale ranges from 1 to 5. Higher values indicate higher gender sensitivity.

n = 155. The data are weighted by level of care and region.

^a^Items were reverse-coded.

M = mean; SD = standard deviation

A thematic analysis of the items reveals particularly strong agreement with statements emphasizing the importance of gender-specific knowledge in medical care. Participants clearly recognized that considering gender differences contributes to improved quality of care and rejected views that such differences are negligible or irrelevant. Lower levels of agreement were observed for statements related to the practical implementation of gender sensitivity in clinical routines. These findings suggest a gap between conceptual awareness and its consistent application in everyday clinical decision-making.

Responses addressing gender in communication and interpersonal interactions showed more heterogeneous patterns, indicating variability in how gender is considered in physician–patient encounters.

Overall, the results point to a generally positive attitude toward gender-sensitive medicine. At the same time, the variability in responses, particularly for items related to practical application, highlights ongoing challenges in translating gender awareness into consistent clinical practice.

### 3.3 Knowledge on gender-specific determinants

Physicians’ knowledge of gender-specific aspects of cardiovascular medicine was assessed using guideline-derived items as described in the Methods section. The overall knowledge score was moderate to high (M = 0.63, SD = 0.08), indicating that, on average, a substantial proportion of the questions were answered correctly. However, considerable variability across items was observed, with correct response rates ranging from 0.39 to 1.00, reflecting heterogeneous levels of topic-specific knowledge within the sample.

While general epidemiological aspects and well-established clinical concepts were widely known, important knowledge gaps emerged in more complex areas, particularly those involving sex-specific diagnostic thresholds and pharmacological differences. For example, only 39% of physicians answered a question correctly about sex-specific ST-segment elevation criteria, and 44% correctly identified sex differences in the interpretation of cardiac biomarkers. In contrast, topics such as symptom presentation and commonly used clinical risk scores were more consistently answered correctly, with correct response rates exceeding 0.80 for several items.

Overall, the variability in performance highlights an uneven distribution of knowledge among physicians. This pattern indicates that current medical training and continuing education may not sufficiently address specific diagnostic and therapeutic implications of sex- and gender-related differences in cardiovascular care (Table 6).

**Table 6:** Physicians’ results on the knowledge questions.

Item	Answer options	Proportion correct answer	n
For which gender is the prevalence of chronic coronary artery disease higher?	**male gender** ◦ female gender ◦ no difference	0.86	139
There is evidence that an exercise ECG generally has lower diagnostic accuracy in women.	**correct** ◦ incorrect	0.83	139
In statin therapy, women without cardiovascular pre-existing conditions experience a slightly lower protective effect than men.	correct **incorrect**	0.67	138
Overall mortality in men and women with known cardiovascular disease is similarly reduced by statins.	**correct** incorrect	0.43	139
Age and gender influence the relative frequency of an acute myocardial infarction with ST-segment elevation (STEMI).	**correct** incorrect	1.00	139
Men and women do not benefit equally from interventional and surgical revascularization therapies.	correct **incorrect**	0.49	138
In clinical practice, ST-segment elevation indicates an acute coronary occlusion. In women, the typical pattern of chest pain shows:	**≥** 1,0 ≥ 1,5 mm ≥ 2,0 mm ≥ 2,5 mm	0.39	138
Compared to men, women have a higher risk of bleeding in antithrombotic treatment.	**correct** incorrect	0.76	139
For stroke risk assessment in atrial fibrillation, I use the CHA2DS2-VASc score and tailor therapy accordingly.	yes I use a different Score: _________________	0.81	139
Female gender is included as a risk factor in the CHA2DS2-VASc score for patients over 65.	**correct** incorrect	0.90	138
In women with myocardial infarction, non-obstructive coronary artery disease or no angiographic evidence of coronary disease occurs up to 30% more often than in men.	**correct** incorrect	0.90	138
Which clinical factors must be considered when evaluating hs-cTn levels in heart attack diagnostics?	**Age** **Renal dysfunction** Liver dysfunction **Time since onset of chest pain** **Sex**	0.44 0.93 0.88 0.87 0.44	139 139 139 139 139
The prevalence of MINOCA is higher in women than in men.	**correct** incorrect	0.84	139
In women, ECG electrodes should be placed as follows:	**under the chest** on the chest	0.86	139
Women with acute myocardial infarction more often present with symptoms such as fatigue, shortness of breath, and nausea.	**correct** incorrect	0.99	138
In addition to classical risk factors, women have further specific risk factors for myocardial ischemia compared to men.	**correct** incorrect	0.78	138
To investigate risk factors in pregnant women in more detail, which of the following guidelines would you consult?	DGK Recommendations for Cardiovascular Wellness in Maternity ESC Guidelines on the Management of Cardiovascular Diseases during Pregnancy ESC Guidelines for Cardiovascular Care in Pregnancy* AWMF Recommendations for cardiovascular health during pregnancy	0.46	121

*Note:* Data are weighted by care level and region. * only this Option exist. The correct answer option is bold and underlined.

#### Perceived implementation of GSC+.

After the knowledge questions physicians were asked to rate the extent to which gender-sensitive care is implemented on their ward. N = 138 Physicians answered this part of the questionnaire. Physicians’ ratings of the perceived implementation of gender-sensitive cardiovascular care (GSC+) are presented in Table 7. Their perceived implementation of GSC + was assessed using the following item:

**Table 7:** Perceived implementation of GSC+.

Group	M	SD	N
Physician	2.45	0.59	138

*“From your perspective, to what extent is gender-sensitive care implemented on this ward (e.g., ensuring patient privacy; respecting religious affiliation, such as providing a space for prayer; addressing migration-related needs, such as language barriers or dietary habits)?”*

Responses were given on a four-point scale ranging from 1 (“Very well implemented”) to 4 (“Not implemented at all”), with an additional “Cannot judge” option.

The average rating among physicians was 2.45 (SD = 0.59), indicating that the implementation of gender-sensitive care is perceived as moderate to low. This perception complements the knowledge results, suggesting a potential gap between awareness of gender-related medical issues and their consistent application in practice. The findings may also reflect a critical professional attitude toward institutional conditions, or a broader lack of prioritization for gender-sensitive care in the medical setting.

## Results of open-ended questions

A qualitative-descriptive summary of the open-text responses is provided to illustrate the variety of perspectives shared by participants.

### 1. The role of gender in physician–patient communication

Participants expressed diverse views on whether and how gender influences clinical communication. Some respondents noted differences in communication styles or expectations depending on patient gender. For example:

“There is a tendency toward more direct communication with male patients, whereas communication with female patients tends to be more cautious and nuanced.”

Other comments highlighted gendered differences in how patients perceive physicians, sensitivity to nonverbal cues, or societal expectations:

“Male physicians are taken more seriously and perceived as more competent, while female physicians, especially in leadership roles, are still often underestimated.”

Several respondents stated that communication should ideally remain gender-neutral and guided by professionalism and empathy. Others pointed to religious or cultural preferences regarding same-gender care providers or privacy needs. Overall, the responses reflect heterogeneous viewpoints, ranging from the belief that gender plays an important role in communication to the view that it should have minimal relevance in everyday practice.

### 2. General remarks on gender-sensitive care

Participants also used the second open-ended item to express concerns about the implementation of gender-sensitive care. Some emphasized that GSC+ remains insufficiently established in clinical routines and requires stronger institutional anchoring. Others referred to persistent gender biases in medical careers:

“As a female physician, you often need to prove yourself twice as much to achieve the same level of recognition.”

A few respondents questioned the extent to which sex and gender differences affect day-to-day decision-making.

Together, these comments illustrate both the perceived relevance of GSC+ and the challenges associated with integrating it into routine clinical practice, though they should be understood as illustrative observations rather than systematically analyzed qualitative findings.

### 3.5 Multivariate analyses

#### Regression analysis of factors associated with physicians’ knowledge.

To identify factors associated with the level of physicians’ knowledge of gender-specific aspects of cardiovascular medicine, a multiple linear regression analysis was conducted. The dependent variable was the average knowledge score, calculated as a mean index across all items (ranging from 0 to 1), with higher values indicating a greater proportion of correct responses. Regression analysis results are shown in Table 8.

**Table 8:** Regression analysis on physicians’ knowledge.

Predictor	Coeff.	Beta	CI (95%)	p-value
University hospital ^a^	0.0375**	0.200	[0.004; 0.067]	0.026
Primary care provider ^a^	0.030	0.167	[-0.002; 0.061]	0.063
R^2^/ Adj. R^2^	0.042/ 0.029			
N	145			

*Note:* Predictor selection was based on regression with backward elimination. Data are unweighted.

^a^Reference category: Tertiary and secondary care providers.

*p < 0.05. **p < 0.01. ***p < 0.001.

A preliminary regression with backward elimination was used to reduce the number of predictors. The initial model included sociodemographic and professional characteristics such as age, gender, country of birth, years of professional experience, medical specialty, and care level of the workplace. Variables with a p-value greater than 0.10 were excluded from the final model.

The final model included two predictors: working in a university hospital (>800 beds) and working in a primary care setting. Both were compared against the reference category of physicians employed in tertiary or secondary care hospitals. The model explained 4.2% of the variance in knowledge scores (R² = 0.042; adjusted R² = 0.029). Physicians working in university hospitals had significantly higher knowledge scores (b = 0.0375, p = 0.026) than those in other hospital types. Physicians in primary care settings also showed a tendency toward higher scores (b = 0.030), although this association did not reach statistical significance (p = 0.063).

These results suggest that individual-level factors such as age, gender, or experience play a minor role in explaining knowledge differences. Instead, structural or institutional contexts particularly the academic setting of university hospitals may foster greater familiarity with gender-specific cardiovascular knowledge, possibly through increased exposure to research, guidelines, or teaching activities.

#### Regression analysis of factors associated with physicians’ gender sensitivity.

To examine which factors are associated with physicians’ gender sensitivity, a multiple linear regression analysis was conducted. The dependent variable was the mean index (scale 1–5) of all items on the gender sensitivity subscale of the N-GAMS questionnaire. Sociodemographic characteristics served as predictors. Predictor selection was based on backward elimination, which retained two variables: country of birth and years of professional experience. The regression models used in the analysis are summarized in Table 9.

**Table 9:** Regression analysis on the gender sensitivity of physicians.

Predictor	Coeff.	Beta	CI (95%)	p-value
Country of Birth (1=DE)	0.375**	0.249	[0.144; 0.607]	0.002
Years of professional experience	0.188	0.144	[-0.013; 0.388]	0.067
R^2^/ Adj. R^2^	0.088/ 0.076			
N	155			

*Note:* Predictor selection was based on regression with backward elimination. Data are unweighted.

*p<0,05. **p<0,01. ***p<0,001.

The final regression model, including these two predictors, explained 8.8% of the variance in gender sensitivity (adjusted R² = 0.076). As shown in Table 9, only the country of birth had a statistically significant effect: physicians born in Germany reported significantly higher gender sensitivity than those born outside of Germany (b = 0.375, p = 0.002). Years of professional experience did not reach statistical significance (p = 0.067).

These findings indicate that sociodemographic characteristics have a limited impact on understanding variations in physicians’ gender sensitivity.

#### Correlation between knowledge, gender sensitivity, and perceived implementation.

To explore the relationships between physicians’ gender sensitivity, knowledge on gender-specific cardiovascular aspects, and their perceived implementation of GSC+ in clinical settings, a correlation analysis was conducted. The correlations between gender sensitivity, knowledge, and perceived implementation of GSC+ are presented in Table 10. As shown, none of the pairwise associations were statistically significant.

**Table 10:** Correlation among physicians.

	Coeff.	p-value	n
Gender sensitivity x knowledge score	0.122	0.177	144
Gender sensitivity x implementation GSC+	-0.092	0.295	0.317
Knowledge score x implementation GSC+	-0.086	0.317	142

*Note:* Data are weighted by level of care and region.

*p < 0.05. **p < 0.01. ***p < 0.001.

There was a weak positive correlation between gender sensitivity (N-GAMS score) and knowledge (r = 0.122, p = 0.177), and a weak negative correlation between gender sensitivity and perceived implementation at the ward level (r = -0.092, p = 0.295). Similarly, knowledge and perceived implementation were unrelated (r = -0.086, p = 0.317). These findings suggest that, in this sample, higher gender sensitivity and knowledge do not necessarily coincide with a higher perception of gender-sensitive practice being implemented.

### 3.6 Hypotheses testing

The hypotheses formulated in Section 2.1 were examined using descriptive statistics, correlation analyses, and multiple regression results. The findings are summarized as follows:

• **H1**: Individual characteristics (e.g., gender, age, migration background, and educational background) influence physicians’ knowledge of gender- and sex-specific cardiovascular care and their level of gender sensitivity.
  → This hypothesis is partially supported. In the regression model predicting knowledge of sex- and gender-specific guideline content, working at a university hospital was a significant positive predictor (b = 0.0375, p = 0.026). Regarding gender sensitivity, physicians born in Germany reported significantly higher scores on the N-GAMS scale compared to those born outside of Germany (b = 0.375, p = 0.002). Other individual factors such as age, gender, and years of professional experience were not significantly associated with either outcome.

- **H2:** Physicians working in urban or higher tier care settings exhibit higher levels of gender sensitivity and knowledge than those in rural or lower tier facilities.
  → This hypothesis is partially supported. Physicians working in university hospitals demonstrated significantly higher knowledge scores. However, no significant association was found between care level or location and gender sensitivity.
- **H3:** Greater knowledge of sex- and gender-specific guideline content is associated with higher gender sensitivity.
  → This hypothesis is not supported. The correlation between knowledge and gender sensitivity was positive but did not reach statistical significance (r = 0.122, p = 0.177; see Table 9).
- **H4:** Perceived GSC+ implementation is positively associated with gender sensitivity.
  → This hypothesis is not supported. No significant relationship was found between gender sensitivity and perceived implementation of gender-sensitive care (r = -0.092, p = 0.295).
- **H5:** There is a gap between the content of clinical guidelines and the actual awareness and implementation of GSC+ in practice.
  → This hypothesis is supported. Although clinical guidelines include substantial gender-specific content, physicians’ knowledge levels were only moderate (M = 0.63, SD = 0.08), and the perceived implementation of gender-sensitive care in daily practice was rated relatively low (M = 2.45, SD = 0.59). This suggests a discrepancy between knowledge of available guidelines and their practical application.

## 4. Discussion

This study examined gender sensitivity and knowledge of GSC+ from guidelines among physicians working in cardiology wards. The findings revealed a quite high level of gender sensitivity (M = 3.95) and moderate knowledge of sex- and gender-specific guideline content for myocardial infarction (M = 0.63).

Regarding the knowledge score, physicians working in university hospitals achieved significantly higher scores compared to those in other care settings. This may be related to a greater exposure to research, academic discussion and ongoing education in the academic setting of university hospitals. The knowledge assessment revealed selective awareness of guideline content. While widely used tools such as the CHA₂DS₂-VASc score were well known, fewer participants were aware of sex-specific recommendations, including diagnostic thresholds or revascularisation indications. This raises questions about the accessibility, visibility and usability of GSC+ content in existing guidelines. In both the survey and prior qualitative phases of the HeartGap study, physicians described gender-related guideline content as addressed selectively, overly technical or the content was only in the appendix of the guideline.The lack of clearly marked or summarized recommendations may hinder practical uptake, particularly in time-pressured environments. Embedding GSC+ into digital decision-support tools could also improve accessibility at the point of care [19]. Currently, there is no binding requirement to integrate sex and gender considerations into clinical guidelines or quality standards in Germany, and similar gaps exist in other countries without formal mandates [20].

In this study, higher knowledge did not translate into greater gender sensitivity or stronger perceptions of GSC+ implementation. The lack of significant correlations between knowledge, gender sensitivity, and perceived implementation suggests a potential gap between cognitive understanding and practical application. Physicians with greater awareness may evaluate implementation more critically, recognizing shortcomings that others may overlook. Conversely, those with limited awareness or knowledge may assume that GSC+ is sufficiently implemented, even when gaps may remain. In addition, perceived implementation may not fully reflect actual practice quality, as it is based on subjective assessment.

In addition, the recruitment strategy may have introduced self-selection bias. As participation was voluntary and based on email invitations and professional networks, physicians with a particular interest in gender-sensitive healthcare may have been more likely to participate. This may have led to an overestimation of gender sensitivity and knowledge levels and limits the generalizability of the findings. These findings should therefore be interpreted as associations rather than evidence of causal relationships between knowledge, gender sensitivity, and perceived implementation.

With regard to gender sensitivity among the sociodemographic variables measured with the N-GAMS scale, only country of birth emerged as a significant predictor. Physicians born in Germany achieved higher gender sensitivity than those born elsewhere. This indicates that cultural socialization and exposure to gender equality norms play an important role in shaping gender-sensitive attitudes. There were no other significant effects for variables such as age, gender, years of professional experience, or gender awareness, which may seem surprising, as it could be expected that these factors influence attitudes toward gender-sensitive care. The assumption that female physicians or those with longer experience are more gender-sensitive was not supported. Those results indicate that gender-sensitive attitudes are shaped less by professional seniority or demographic background and but also by personal reflection, targeted training experiences and broader institutional influences.

The N-GAMS was originally developed for medical students and later adapted for use with health professionals. Few studies have applied N-GAMS in physician populations, particularly in Germany. Evidence from other countries indicates that gender sensitivity is shaped by broader factors than individual characteristics alone. In France, a study among general practitioners found that the French N-GAMS demonstrated high internal consistency, a stable factor structure consistent with the original subscales, and expected correlations with related constructs, indicating that it is reliably and validly measures gender sensitivity and gender-role attitudes in this setting. The findings also showed that demographic and professional variables explained only part of the variance, highlighting the importance of cultural and educational context [21]. Similarly, the Arabic version, validated among primary care physicians and nurses in Palestine, showed acceptable reliability and revealed demographic variations in gender sensitivity, while also pointing to structural and sociocultural factors as likely determinants [8].The Portuguese N-GAMS was adapted and validated with a large sample of medical students (n = 1,048), confirming the original three-factor structure (Gender Sensitivity, Gender-Role Ideology toward Patients, Gender-Role Ideology toward Doctors) with good internal consistency and expected associations with empathy and sexist attitudes, supporting both construct and criterion validity [22]. These international findings also indicate that gender-sensitive attitudes and behaviors are embedded within broader educational, cultural, and organizational contexts.

The responses to the open-ended questions further illustrated the perceived role of gender in physician–patient communication. Several participants described differences in communication style or expectations depending on the patient’s gender, such as more cautious or emotionally attuned communication with women and more direct approaches with men. Others argued that communication should be guided primarily by empathy and professional standards rather than gender. Persistent biases were noted, including the perception that male physicians are taken more seriously or that some patients (mostly female patients) even prefer male doctors. Such views reflect entrenched stereotypes and stand in contrast to evidence, e.g., from a large retrospective study of over 580,000 heart attack cases in Florida, which found that female patients treated by female physicians had significantly higher survival rates than those treated by male physicians. In that study, the gender gap in outcomes among female patients was substantially reduced when they were cared for by female doctors [23]. These discrepancies suggest the complex relation between perception, behaviour, and clinical effectiveness.

In a prior part of the HeartGap Study, a scoping review was conducted to reveal barriers and facilitators to implement gender sensitive care, and also in which domains it should be implemented [24]. The review revealed that the implementation of GSC + is a holistic topic. It emphasizes the need for binding political frameworks and targeted funding, the mandatory integration of sex and gender analysis in research, the inclusion of GSC+ content in medical and nursing curricula, and organizational strategies in healthcare institutions such as quality circles or change agents. Overall, GSC+ should be anchored holistically across politics, research, education, and care institutions [24].

Although physicians showed a generally high level of gender sensitivity, their knowledge of guideline content was only moderate, revealing marked gaps in areas such as diagnostic thresholds and pharmacological sex differences. Moreover, higher knowledge did not translate into more positive perceptions of GSC+ implementation, underlining the persistent gap between awareness and practice. The fact that physicians in university hospitals achieved higher knowledge scores suggests that institutional context plays a role, while the influence of individual factors was limited. These results indicate that gender-sensitive care is shaped less by professional seniority or demographics and more by institutional context and training opportunities.

The findings of this study also carry practical implications for clinical care. The identified knowledge gaps, particularly regarding sex-specific diagnostic thresholds, symptom patterns, and treatment recommendations, highlight concrete areas for optimizing clinical decision-making. Improving the visibility and clarity of GSC+ recommendations in guidelines, embedding relevant thresholds into digital decision-support tools, and incorporating targeted training modules could help physicians make more accurate, timely, and individualized diagnostic and therapeutic decisions. Such measures may help to reduce diagnostic delays, enhance treatment precision, and ultimately improve patient outcomes, especially for women and gender-diverse individuals who are disproportionately affected by cardiovascular misdiagnosis [25].

To ensure equitable, evidence-based care, gender-sensitive principles should therefore be embedded as a binding component of guideline development, postgraduate training, and institutional quality frameworks. This would require clearly identifiable sections in clinical guidelines, not only in appendices, along with measurable quality indicators and integration into specialist training regulations [26,27]. These implications are consistent with and reinforced by findings from the prior scoping review, which emphasized the need for political frameworks, targeted funding, curricular integration, and organizational strategies [24]. Taken together, the empirical findings of this study and the broader review underscore that implementing GSC+ must be approached holistically across politics, research, education, and healthcare institutions.

Future research should explore how institutional culture, interprofessional collaboration and leadership may influence the translation of GSC+ knowledge into daily practice. Longitudinal studies could help clarify how gender sensitivity evolves over time and identify which educational, organizational, or policy interventions are most effective. Closing the gap between policy and practice will require coordinated efforts by educators, professional societies and policymakers at both national and international levels.

## Limitations

This study has some limitations that should be considered when interpreting the findings. The cross-sectional design captures data at a single point in time and therefore precludes causal inferences regarding the relationships between knowledge, gender sensitivity, and the perceived implementation of GSC + . As such, the observed associations should be interpreted as descriptive rather than indicative of underlying causal mechanisms.

The use of a self-administered online survey introduces several potential sources of bias. Given the reliance on self-reported measures, responses may be influenced by social desirability and subjective overestimation, particularly in relation to gender sensitivity and the perceived implementation of GSC + . In addition, self-selection bias cannot be excluded, as physicians with a pre-existing interest in GSC+ may have been more likely to participate. This may have resulted in a sample that is systematically more aware of, and engaged with, gender-related issues than the broader physician population, thereby potentially inflating estimates of knowledge and sensitivity.

Methodological constraints related to measurement further qualify the interpretation of the findings. The knowledge assessment was based on selected, self-developed items derived from current cardiology guidelines. While content validity was supported through systematic guideline extraction and expert review, the instrument does not capture the full scope and complexity of sex- and gender-specific cardiovascular knowledge and requires further psychometric validation. Similarly, perceived implementation was assessed using a single-item indicator, which limits reliability and does not adequately reflect the multidimensional and context-dependent nature of implementation processes. The measure captures a general subjective impression and does not allow for differentiation between specific domains, organizational levels, or structural determinants of implementation.

Although the sample size was sufficient for descriptive and regression analyses, it constrains the robustness of subgroup comparisons, particularly with regard to institutional characteristics. Smaller subgroup sizes may have limited statistical power to detect meaningful differences.

Finally, as the study was conducted within the German healthcare system, the findings need to be interpreted in light of its specific structural and institutional characteristics. These include a comparatively high degree of standardization through national and European clinical guidelines, as well as particular features of postgraduate medical training and hospital organization. In addition, the integration of sex- and gender-specific content into clinical practice is likely influenced by country-specific educational curricula, policy priorities, and professional norms. Against this background, the observed patterns of knowledge, gender sensitivity, and implementation may not readily translate to other healthcare systems, where structural conditions, resource allocation, and the prioritization of GSC+ differ substantially

## 5. Conclusion

This study provides new evidence on the awareness and implementation of GSC+ in German inpatient cardiology. Physicians demonstrated a generally high level of gender sensitivity, but only moderate knowledge of sex- and gender-specific guideline content, with notable gaps regarding diagnostic thresholds and pharmacological sex differences. Knowledge levels were higher in university hospitals, indicating an influence of institutional context, while gender sensitivity was associated with country of birth, suggesting the role of cultural socialization and exposure to equality norms. However, greater knowledge did not translate into higher gender sensitivity, indicating a gap between awareness and practice.

The findings point to several implications for practice, science, and policy. To make guideline content more usable, sex- and gender-specific recommendations should be presented more visibly within the main sections of clinical guidelines and embedded into digital decision-support systems. Moreover, as artificial intelligence (AI) applications are increasingly integrated into clinical decision-making, it is essential that these systems are trained on sex- and gender-differentiated data. Without such stratification, algorithms risk perpetuating existing biases.

Medical training and continuing education should incorporate GSC+ as a mandatory component to ensure that physicians are adequately prepared to apply this knowledge in daily care. Institutional strategies, including measurable quality indicators and structured training frameworks, are essential to foster gender-sensitive practices beyond individual commitment. Strengthening interdisciplinary collaboration between physicians, nurses, and other healthcare professionals is also crucial, as effective implementation of GSC+ requires coordinated perspectives and shared responsibilities across professional groups.

Beyond institutional change, the findings underline the relevance of GSC+ for patient outcomes. Better awareness of sex- and gender-specific diagnostic criteria and treatment strategies can help reduce misdiagnosis, inappropriate therapy, and outcome disparities. This suggests that GSC + is not merely a matter of professional attitudes but a prerequisite for high-quality, equitable, and evidence-based care.

**Fig 3:**
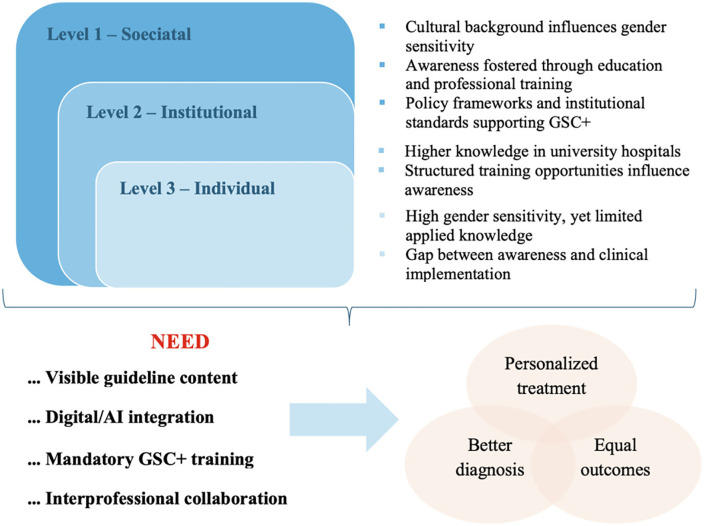
Summary of key results on gender sensitivity, knowledge, and GSC+ implementation.

These findings further emphasize the practical need to integrate sex- and gender-specific knowledge into everyday clinical routines by strengthening guideline usability, enhancing digital decision-support, and establishing institutional structures that promote consistent GSC+ application. By translating evidence into accessible tools and supportive environments, cardiology departments can more effectively bridge the gap between awareness and practice. Fig 3 provides an overview of the key study results.

Taken together, these results implementing GSC+ requires coordinated action at multiple levels, from guideline development and education to institutional culture, interprofessional collaboration, and policy frameworks. Only through such an integrated approach can gender-sensitive cardiovascular care move from being a recommended ideal to becoming a consistent reality in everyday clinical practice.

## Supporting information

S1 Questionnaire Physicians´s questionnaire (English translation). (DOCX)

S1 Text Summary of sex- and gender- specific differentiations in cardiovascular guidelines. (DOCX)

## Abbreviations

AWMF: Arbeitsgemeinschaft der Wissenschaftlichen Medizinischen Fachgesellschaften (Association of the Scientific Medical Societies in Germany)
CHA₂DS₂-VASc: Clinical risk score for stroke prediction in atrial fibrillation (Congestive heart failure, Hypertension, Age ≥ 75 years, Diabetes, Stroke/TIA, Vascular disease, Age 65–74 years, Sex category)
DGK: Deutsche Gesellschaft für Kardiologie (German Cardiac Society)
DRKS: Deutsches Register Klinischer Studien (German Register of Clinical Studies)
ESC: European Society of Cardiology
GSC+: Gender-sensitive care
GSNC+: Gender-sensitive personalized nursing care plus
Habil.: Habilitation (postdoctoral qualification)
HS-cTn: High-sensitivity cardiac troponin
MHH: Medizinische Hochschule Hannover (Hannover Medical School)
MINOCA: Myocardial Infarction with Non-Obstructive Coronary Arteries
N-GAMS: Nijmegen Gender Awareness in Medicine Scale
STEMI: ST-Segment Elevation Myocardial Infarction
WHO: World Health Organization.
