# Supplementary material for: From evidence to action: Gender-sensitive cardiovascular care – A quantitative survey with physicians working in cardiology departments
Source: PLOS Glob Public Health. 2026 Apr 29;6(4):e0006357. doi: 10.1371/journal.pgph.0006357 (PMC13127897; doi:10.1371/journal.pgph.0006357)
Supplement: S1 Questionnaire — (DOCX) [file pgph.0006357.s001.docx]

Dear participant,

Thank you for your willingness to take part in the survey!

As part of the HeartGap project funded by the Innovation Fund, we would like to ask you about your views and experiences on the topic of gender-sensitive medical care.

Hannover Medical School (MHH) and the Private Research Institute for Health and System Design (figus GmbH) have been commissioned by the Joint Federal Committee to carry out the evaluation. The study is registered at with the German Register of Clinical Studien (DRKS).

The survey takes about 10 minutes. We assure you that all your data will be analysed anonymously and that no conclusions can be drawn about you personally. The findings will be used to determine the status quo of gender-sensitive care. The data will not be passed on to third parties. Anonymised data will be published in specialist publications.

Please complete the questionnaire as fully as possible and use

as a guide for your initial thoughts. Thank you for your participation!

**Do you have any questions?**

Sophia Sgraja, M.A. (Research Associate, Institute of Epidemiology,

Social Medicine and Health Systems Research, Hannover Medical School)

E-Mail: Sgraja.Sophia@mh-hannover.de

Tel.: +49 511 532-5417

| **PART A:** Socio-demographic data  *Please answer the following questions about yourself.* | | | | | | | | | | |
| --- | --- | --- | --- | --- | --- | --- | --- | --- | --- | --- |
| **A1. Gender:** |  | | | | | | | | | |
| Ο | male | | | | | | | | | |
| Ο | female | | | | | | | | | |
| Ο | diverse | | | | | | | | | |
|  |  | | | | | | | | | |
| **A2. Age (in years):** |  | | | | | | | | | |
|  |  | | | | | | | | | |
|  |  | | | | | | | | | |
|  |  | | | | | | | | | |
|  |  | | | | | | | | | |
| **A3. Born in Germany?** | | | | | | | | | | |
| Ο | Yes | | | | | | | | | |
| Ο | No | | | | | | | | | |
|  |  | | | | | | | | | |
| **A4. Highest school-leaving qualification:** | | | | | | | | | | |
| Ο | (Fach-) Gymnasium/(Fach)-Abitur/comparable | | | | | | | | | |
| Ο | Mittlere Reife/ Realschule/comparable | | | | | | | | | |
| Ο | Hauptschulabschluss | | | | | | | | | |
| Ο | No qualification | | | | | | | | | |
| Ο | Other: | | | | | | | | | |
|  |  | | | | | | | | | |
|  |  | | | | | | | | | |
|  |  | | | | | | | | | |
| **A5. Highest subject-specific professional qualification:** | | | | | | | | | | |
| Ο | Habilitation | | | | | | | | | |
| Ο | Doctorate | | | | | | | | | |
| Ο | (Professional) university degree (Bachelor's, Master's, state examination) | | | | | | | | | |
| Ο | Completed vocational training | | | | | | | | | |
| Ο | Currently in training / studying | | | | | | | | | |
| Ο | Without a vocational qualification | | | | | | | | | |
| Ο | Other: | | | | | | | | | |
|  |  | | | | | | | | | |
| **A6. Employed in the medical profession since (incl. training, in years):** | | | | | | | | | | |
|  |  | | | | | | | | | |
|  |  | | | | | | | | | |
|  |  | | | | | | | | | |
|  |  | | | | | | | | | |
| **A7. Size of the institution in which you work:** | | | | | | | | | | |
| Ο | University hospital (>800 beds & teaching/research) | | | | | | | | | |
| Ο | Maximum care (>800 beds) | | | | | | | | | |
| Ο | Specialist care (501-800 beds) | | | | | | | | | |
| Ο | Basic care (<=500 beds) | | | | | | | | | |
| Ο | University hospital (>800 beds & teaching/research) | | | | | | | | | |
|  |  | | | | | | | | | |
| **A8. Region of the institution:** | | | | | | | | | | |
| Ο | Large city (>100,000 inhabitants) | | | | | | | | | |
| Ο | City (5,000-100,000 inhabitants) | | | | | | | | | |
| Ο | Rural municipality (<5,000 inhabitants) | | | | | | | | | |
|  |  | | | | | | | | | |
|  |  | | | | | | | | | |
|  |  | | | | | | | | | |
|  |  | | | | | | | | | |
| **PART B:** Medical guidelines in healthcare practice | | | | | | | | | | |
| **B1. Which of the following guidelines do you use in your care practice?** | | | | | | | | | | |
| Ο | Association of the Scientific Medical Societies in Germany (AWMF): S3 Guideline National Care Guideline Chronic CHD | | | | | | | | | |
| Ο | German Society of Cardiology (DGK): Acute Coronary Syndrome without ST- segment elevation (NSTE-ACS) | | | | | | | | | |
| Ο | German Society of Cardiology (DGK): Therapy of acute myocardial infarction in patients with ST-segment elevation (STEMI) | | | | | | | | | |
| Ο | European Society of Cardiology (ESC) Guideline: 2020 Acute Coronary Syndromes (ACS) in Patients without Persistent ST-Segment Evaluation (Management of) Guidelines | | | | | | | | | |
| Ο | European Society of Cardiology (ESC) Guideline: 2023 ESC Guideline for the management of acute coronary syndromes. | | | | | | | | | |
| Ο | None | | | | | | | | | |
| Ο | Other: | | | | | | | | | |
|  |  | | | | | | | | | |
|  |  | | | | | | | | | |
|  |  | | | | | | | | | |
| **PART C:** Attitudes towards gender sensitivity in medical care | | | | | | | | | | |
| **C1. Various statements on "Gender differences in medicine" are listed below. Please assess how well this statement applies to you personally and tick the box.** | | | | | | | | | | |
|  | | **I completely disagree** | | **I mostly disagree** | | **I partly agree/ partly disagree** | | **I mostly agree** | **I completely agree** | |
| Physicians' in-depth knowledge of gender differences improves the quality of medical care. | | Ο | | Ο | | Ο | | Ο | Ο | |
| Physicians should only deal with biological differences between men* and women* | | Ο | | Ο | | Ο | | Ο | Ο | |
| In the case of non-gender-specific complaints, the gender of the patient is irrelevant. | | Ο | | Ο | | Ο | | Ο | Ο | |
| Physicians should limit themselves as far as possible to purely medical aspects of health complaints of men* and women*. | | Ο | | Ο | | Ο | | Ο | Ο | |
| Physicians do not need to know what is going on in the lives of men* and women* in order to provide medical care. | | Ο | | Ο | | Ο | | Ο | Ο | |
| Differences between female physicians and male physicians are too small to be relevant. | | Ο | | Ο | | Ο | | Ο | Ο | |
| Physicians should treat all patients equally precisely because men* and women* are different. | | Ο | | Ο | | Ο | | Ο | Ο | |
| Physicians who deal with gender differences do not deal with the important issues. | | Ο | | Ο | | Ο | | Ο | Ο | |
| When communicating with patients, it does not matter to the doctors whether the patients are male or female. | | Ο | | Ο | | Ο | | Ο | Ο | |
| When communicating with patients, it does not matter whether the doctor is male or female. | | Ο | | Ο | | Ο | | Ο | Ο | |
| Differences between male and female  patients are so small that physicians  can hardly take them into account. | | Ο | | Ο | | Ο | | Ο | Ο | |
| Physicians should address gender differences in the causes and consequences of disease for effective treatment. | | Ο | | Ο | | Ο | | Ο | Ο | |
| It is not necessary to take gender differences into account when describing complaints to. | | Ο | | Ο | | Ο | | Ο | Ο | |
| **PART D:** Knowledge about gender-specific care from the medical guidelines | | | | | | | | | | |
| **D1. Which gender has a higher prevalence of chronic coronary heart disease?** | | | | | | | | | | |
| Ο | male | | | | | | | | | |
| Ο | female | | | | | | | | | |
| Ο | no difference | | | | | | | | | |
|  |  | | | | | | | | | |
| **D2. There are indications that an exercise ECG generally has a lower diagnostic value in women.** | | | | | | | | | | |
| Ο | Correct | | | | | | | | | |
| Ο | Incorrect | | | | | | | | | |
|  |  | | | | | | | | | |
| **D3. Statin therapy has a slightly lower protective effect in women without underlying cardiovascular disease than in men.** | | | | | | | | | | |
| Ο | Correct | | | | | | | | | |
| Ο | Incorrect | | | | | | | | | |
|  |  | | | | | | | | | |
| **D4. Overall mortality in men and women with known cardiovascular disease is reduced by statins to a similar extent.** | | | | | | | | | | |
| Ο | Correct | | | | | | | | | |
| Ο | Incorrect | | | | | | | | | |
|  |  | | | | | | | | | |
| **D5. Age and gender have an influence on the relative frequency of an acute ST-segment elevation myocardial infarction (STEMI).** | | | | | | | | | | |
| Ο | Correct | | | | | | | | | |
| Ο | Incorrect | | | | | | | | | |
|  |  | | | | | | | | | |
| **D6. Women and men do not benefit equally from interventional and surgical reperfusion therapies.** | | | | | | | | | | |
| Ο | Correct | | | | | | | | | |
| Ο | Incorrect | | | | | | | | | |
|  |  | | | | | | | | | |
|  |  | | | | | | | | | |
|  |  | | | | | | | | | |
|  |  | | | | | | | | | |
| **D7. In the clinical context, ST-segment elevation indicates an acute occlusion of the coronary artery. For women in the chest wall leads, a normal value of:** | | | | | | | | | | |
| Ο | > 1.0 mm | | | | | | | | | |
| Ο | >1.5 mm | | | | | | | | | |
| Ο | >2.0 mm | | | | | | | | | |
| Ο | >2.5 mm | | | | | | | | | |
|  |  | | | | | | | | | |
| **D8. Compared to men, women may have a higher risk of bleeding during antithrombotic treatment.** | | | | | | | | | | |
| Ο | Correct | | | | | | | | | |
| Ο | Incorrect | | | | | | | | | |
|  |  | | | | | | | | | |
| **D9. I use the CHA2DS2-VASc score to assess the risk of a stroke in atrial fibrillation and adjust the therapy accordingly.** | | | | | | | | | | |
| Ο | Yes | | | | | | | | | |
| Ο | I use a different score: | | | | | | | | | |
|  |  | | | | | | | | | |
|  |  | | | | | | | | | |
|  |  | | | | | | | | | |
|  |  | | | | | | | | | |
| **D10. Female gender is included in the CHA2DS2-VASc score as a risk factor.** | | | | | | | | | | |
| Ο | Correct | | | | | | | | | |
| Ο | Incorrect | | | | | | | | | |
|  |  | | | | | | | | | |
| **D11. Women with myocardial infarction are up to 30% more likely to have non-stenosing coronary atherosclerosis or no angiographic evidence of coronary heart disease compared to men.** | | | | | | | | | | |
| Ο | Correct | | | | | | | | | |
| Ο | Incorrect | | | | | | | | | |
|  |  | | | | | | | | | |
| **D12. Which clinical influencing factors have to be considered when assessing the concentration of hs-cTn in myocardial infarction diagnostics?** | | | | | | | | | | |
| Ο | Age | | | | | | | | | |
| Ο | Renal dysfunction | | | | | | | | | |
| Ο | Liver dysfunction | | | | | | | | | |
| Ο | Time since onset of chest pain | | | | | | | | | |
| Ο | Gender | | | | | | | | | |
|  |  | | | | | | | | | |
|  |  | | | | | | | | | |
| **D13. The prevalence of MINOCA is higher in women than in men.** | | | | | | | | | | |
| Ο | Correct | | | | | | | | | |
| Ο | Incorrect | | | | | | | | | |
|  |  | | | | | | | | | |
| **D14. For women, the ECG electrodes should be positioned as follows:** | | | | | | | | | | |
| Ο | Under the chest | | | | | | | | | |
| Ο | On the chest | | | | | | | | | |
|  |  | | | | | | | | | |
| **D15. Women with acute myocardial infarction more frequently show symptoms such as abnormal tiredness, shortness of breath and nausea.** | | | | | | | | | | |
| Ο | Correct | | | | | | | | | |
| Ο | Incorrect | | | | | | | | | |
|  |  | | | | | | | | | |
| **D16. In addition to the classic risk factors, women have additional risk factors for myocardial ischemia compared to men.** | | | | | | | | | | |
| Ο | Correct | | | | | | | | | |
| Ο | Incorrect | | | | | | | | | |
|  |  | | | | | | | | | |
| **D17. For more in-depth information on risk factors in pregnant women, which of the following guidelines would you use?** | | | | | | | | | | |
| Ο | DGK Recommendations for Cardiovascular Wellness in Maternity | | | | | | | | | |
| Ο | ESC Guidelines on the Management of Cardiovascular Diseases during Pregnancy | | | | | | | | | |
| Ο | ESC Guidelines for Cardiovascular Care in Pregnancy | | | | | | | | | |
| Ο | AWMF Recommendations for Cardiovascular Health in Pregnancy | | | | | | | | | |
|  |  | | | | | | | | | |
| **D18. Please answer the final question:** | | | | | | | | | | |
|  | | Very good | Good | | Limited | | Not imple-mented | | | I cannot judge |
| In your opinion, to what extent is gender-sensitive care implemented on this ward?  (e.g. maintaining patients’ privacy; respecting religious affiliation, such as providing space for prayer; or considering migration background, such as language barriers or dietary habits) | | Ο | Ο | | Ο | | Ο | | | Ο |
|  |  | | | | | | | | | |
|  |  | | | | | | | | | |
|  |  | | | | | | | | | |
| **D19. Optional: In your opinion, what role does the patient's gender play in physician-patient communication?** | | | | | | | | | | |
|  |  | | | | | | | | | |
|  |  | | | | | | | | | |
|  |  | | | | | | | | | |
|  |  | | | | | | | | | |
|  |  | | | | | | | | | |
|  |  | | | | | | | | | |
|  |  | | | | | | | | | |
|  |  | | | | | | | | | |
|  |  | | | | | | | | | |
| **D20. Is there anything else you would like to say on the subject?** | | | | | | | | | | |
| *Notes* (optional): |  | | | | | | | | | |
|  |  | | | | | | | | | |
|  |  | | | | | | | | | |
|  |  | | | | | | | | | |
|  |  | | | | | | | | | |
|  |  | | | | | | | | | |
|  |  | | | | | | | | | |
|  |  | | | | | | | | | |
|  |  | | | | | | | | | |
|  |  | | | | | | | | | |
|  |  | | | | | | | | | |
|  |  | | | | | | | | | |
|  |  | | | | | | | | | |
|  |  | | | | | | | | | |
|  |  | | | | | | | | | |
|  |  | | | | | | | | | |
|  |  | | | | | | | | | |
|  |  | | | | | | | | | |
|  |  | | | | | | | | | |
|  |  | | | | | | | | | |

**Thank you very much for your participation!**

If you are interested in a summary of the results of the last part of the

content on gender-sensitive care in the guidelines, please send an

informal e-mail to: [Sgraja.Sophia@mh-hannover.de](mailto:Sgraja.Sophia@mh-hannover.de)

or contact by telephone on: +49 511 532-5417.

**Sophia Sgraja, M.A.** (Research Associate, Institute of Epidemiology,

Social Medicine and Health Systems Research, Hannover Medical School)
