# Supplementary material for: From evidence to action: Gender-sensitive cardiovascular care – A quantitative survey with physicians working in cardiology departments
Source: PLOS Glob Public Health. 2026 Apr 29;6(4):e0006357. doi: 10.1371/journal.pgph.0006357 (PMC13127897; doi:10.1371/journal.pgph.0006357)
Supplement: S1 Text — (DOCX) [file pgph.0006357.s002.docx]

**Gender-specific Differentiation in Medical Guidelines**

Authors: Sophia Sgraja^a^, Frederike Wiese^a^ Judith Mollenhauer^b^, Prof. Dr. Volker E. Amelung^a^, Prof. Dr. Clarissa Kurscheid^a^, Prof. Dr. Ute Seeland^c^

^a^ Institute for Epidemiology, Social Medicine, and Health System Research, Hannover Medical School

^b^ figus GmbH – Private Research Institute for Health and System Design

^c^ Faculty of Medicine, Otto von Guericke University Magdeburg, Section for Gender-Sensitive Medicine

The HeartGap project (funding code 01VSF22030) is a healthcare research study funded by the Innovation Fund with a funding period from January 1, 2023, to June 30, 2025. The consortium consists of the private research institute for health and system design (figus GmbH) and Hannover Medical School. The project is supported in terms of content and communication by the Institute for Gender Health e.V. and Prof. Dr. Ute Seeland (Chair of Internal Medicine/Gender-Sensitive Medicine at Otto von Guericke University in Magdeburg).

The acronym HeartGap stands for gender health gaps in guideline-oriented inpatient cardiological care and implementation strategies for reducing them. It examines the extent to which evidence-based, gender-sensitive, personalized medicine and care is implemented in cardiology wards and what needs patients have in this regard.

Brief overview

The gender of patients and doctors can affect different parts of medical care and communication. Gender-sensitive care is an important step in medical practice to meet the individual needs of patients. Gender encompasses both biological sex (female, male, intersex) and social gender (gender roles, characteristics, and behaviors defined by society). In addition, other diversity factors such as age, migration background, religious affiliation, sexual orientation, and physical and psychological characteristics are important for the personalized treatment of patients.

The following summary is intended for physicians and provides guidance on implementing gender-sensitive care based on the AWMF guidelines of the Association of Scientific Medical Societies¹, guidelines of the German Society of Cardiology (DGK)², and selected ESC guidelines⁴. These guidelines were developed through systematic literature reviews and expert knowledge and include gender-specific aspects as well as other diversity factors relevant to medical care.

The focus of this analysis was on the field of cardiology, which is why cardiological guidelines focusing on myocardial infarction were analyzed for gender-specific differences. The AWMF guidelines also contain important definitions of terms relating to gender, women, men, female, male, and other diversity factors. The following table presents gender- and diversity-sensitive content as well as practice-oriented measures that were developed based on the aforementioned guidelines. The first column shows the underlying question from the survey, the second column contains the possible answers, with the row marked in green indicating the correct answer. The last column contains specific recommendations for gender-sensitive medical care.

The evaluation refers to key cardiological conditions, including chronic coronary heart disease (CHD), acute myocardial infarction with ST-segment elevation (STEMI), acute coronary syndrome without ST-segment elevation (NSTEMI), atrial fibrillation, and other acute coronary syndromes (ACS).

| **Question** | **Possible answers** | **Reference in Guideline** |
| --- | --- | --- |
| **AWMF**  *S3 guideline on chronic CHD***^[[1]](#endnote-2)^** | | |
| **Which gender has a higher prevalence of chronic cardiovascular coronary heart disease?** |  male gender   female gender   no difference | **See section 2.2; page 14**  In addition to male gender and older age, low social status is also associated with a higher lifetime prevalence of CHD (including heart attack) (see Table 2). |
| **There is evidence that stress ECGs are generally less diagnostically significant in women.** |  correct   incorrect | **See section 4.1.5.1; page 25**  Two meta-analyses showed a generally lower diagnostic significance in women. The exercise ECG is not meaningful in cases of insufficient physical fitness, WPW, ventricular rhythm, ST depression > 1 mm at rest, and complete left bundle branch block [65]. The diagnostic significance is also limited in women, in cases of digitalis, nitrate, or beta-blocker use, and in cases of ST segment depression of 0-1 mm at rest. |
| **In statin therapy, even after correcting for non-gender-specific differences, women without underlying cardiovascular disease have a slightly lower protective effect than men.** |  correct   incorrect | **See section 7.2.1., page 60**  With regard to the composite endpoint of major cardiovascular events (cardiovascular death, non-fatal myocardial infarction, coronary revascularization, stroke), the effectiveness differed depending on cardiovascular risk: In women without underlying cardiovascular disease, the protective effect was slightly lower than in men, even after correction for non-gender-specific differences. In women and men with known cardiovascular disease or a 5-year risk of cardiovascular events > 20%, statins had a similar effect on the reduction of serious cardiovascular events. |
| **The overall mortality rate in men and women is reduced by statins to a similar extent.** |  correct   incorrect | **See section 7.2.1., page 60**  With regard to the composite endpoint of major cardiovascular events (cardiovascular death, non-fatal myocardial infarction, coronary revascularization, stroke), the effectiveness differed depending on cardiovascular risk: In women without underlying cardiovascular disease, the protective effect was slightly lower than in men, even after correction for non-gender-specific differences. In women and men with known cardiovascular disease or a 5-year risk of cardiovascular events > 20%, statins had a similar effect on the reduction of serious cardiovascular events. |
| **German Society of Cardiology (DGK)**  *Treatment of acute myocardial infarction in patients with ST-segment elevation (STEMI)^[[2]](#endnote-3)^* | | |
| **Age and gender influence the relative frequency of acute myocardial infarction with ST-segment elevation (STEMI).** |  correct   incorrect | **See section 1.2**  The relative frequency of STEMI is decreasing and that of NSTEMI is increasing. There is a consistent pattern for STEMI, namely that it occurs relatively more frequently in younger than in older people and more frequently in men than in women. |
| **Women and men do not benefit equally from interventional and surgical reperfusion therapies.** |  correct   incorrect | **See section 2.1**  These guidelines are intended to make it clear that women and men benefit equally from reperfusion and STEMI-related therapies and that both sexes must be treated in a similar manner. |
| **In a clinical context, ST segment elevation indicates acute coronary artery occlusion. The normal value for women is:** |  -≥1,0   ≥1,5 mm   -≥ 2,0 mm   -≥ 2,5 mm | In the appropriate clinical context, ST segment elevation (measured at the J point) is considered an indication of persistent acute coronary artery occlusion in the following cases: ≥2 contiguous leads with ST segment elevation ≥2.5 mm in men <40 years of age, ≥2 mm in men ≥40 years of age, or ≥1.5 mm in women in leads V2-V3 and/or ≥1 mm in the other leads. |
| **German Society of Cardiology (DGK)**  *Treatment of acute myocardial infarction in patients without ST-segment elevation (STEMI)* | | |
| **Compared to men, women may have a higher risk of bleeding when undergoing antithrombotic treatment.** |  correct   incorrect | **See section 4.3, page 30f and section 5.3, page 50**  Women may have an increased risk of bleeding compared to men when receiving antithrombotic treatment for coronary heart disease. This could be due to various gender-specific and individually variable factors. Women show differences in the pharmacokinetics and pharmacodynamics of antithrombotic drugs, influenced by aspects such as body composition, fat percentage, and hormonal fluctuations, particularly in the menstrual cycle.  Furthermore, there is evidence that women may generally have an increased tendency to bleed due to biological factors and hormonal influences. This predisposition could manifest itself in an increased risk of bleeding complications during antithrombotic therapy. Hormonal changes in the female life cycle, such as pregnancy and menopause, can also influence the risk of bleeding. The interaction between estrogens and blood coagulation factors plays a particularly important role here. |
| **I use the CHA2DS2-VASc score to assess the risk of stroke in atrial fibrillation and tailor the treatment accordingly.** |  Yes   I use a different one  Score: ___________ | **Information text following the question below**  The 2024 ESC guidelines recommend a revised version of the score (CHA₂DS₂-VA score), which no longer includes the category “female gender” (“Sc”).^4^ |
| **Being female is included as a risk factor in the CHA2DS2-VASc score for patients over 65.** |  correct   incorrect | **See section 5.3, page 55**  The CHA2DS2-VASc score is the recommended method for assessing the risk of stroke in atrial fibrillation. This score allows for a differentiated consideration of various risk factors in order to more accurately assess individual stroke risks and thus determine the need for anticoagulant therapy.  With regard to female gender, the score takes into account the fact that women over the age of 65 have a slightly higher risk of stroke in atrial fibrillation than men. This gender-specific aspect is taken into account by assigning points for female gender in the score. In addition to gender and age, the score also takes into account the presence of existing vascular disease by assigning different scores depending on age group. ^[[3]](#endnote-4)^ |
| **In women, non-stenotic coronary atherosclerosis or no angiographic evidence of coronary heart disease may be present in approximately 30% more cases than in men following a heart attack.** |  correct   incorrect | Approximately 30% of women who suffer a heart attack are more likely to have non-stenotic coronary atherosclerosis or no angiographic evidence. This underscores the importance of considering gender-specific differences in the diagnosis and treatment of heart disease. The presence of non-stenotic coronary atherosclerosis, which may not be clearly visible in angiographic examinations, suggests that women may develop other forms of vascular changes that may not be detected by traditional angiographic examinations. It is particularly important in women and older patients to adjust the dosage of anticoagulants to body weight and kidney function. |
| **What clinical factors should be considered when assessing hs-cTn concentration in the diagnosis of myocardial infarction?** |  age   renal dysfunction   liver dysfunction   Time since onset of chest pain   gender |  |
| **ESC Guideline**  *2020 Acute Coronary Syndromes (ACS) in Patients Presenting without Persistent ST-Segment Elevation (Management of)* Guidelines^[[4]](#endnote-5)^ | | |
| **The prevalence of Minoca is higher in women.** |  correct   incorrect | Patients with MINOCA (myocardial infarction without coronary obstruction) are more likely to be female than patients with NSTE-ACS (non-ST-elevation myocardial infarction with coronary obstruction). MINOCA refers to cases of heart attack where no obvious narrowing of the coronary arteries is detected, in contrast to NSTE-ACS, where such obstruction is detectable.  The prevalence of MINOCA is higher in women, and this may be due to gender-specific differences in the pathophysiology and presentation of cardiovascular disease.  **See section 7; page 1329**  Compared with patients with obstructive CAD, NSTE-ACS patients diagnosed with MINOCA are more likely to be younger and female, and less likely to be diabetic, hypertensive, or dyslipidaemic, suggesting a predominant role of non-atherosclerotic-related aetiologies and of unusual or usual risk factors such as psychosocial aspects, insulin resistance, and inflammation. |
| **Additional questions:** Not from guidelines | | |
| **In women, the ECG electrodes should be positioned as follows.** |  | Correct electrode placement depends on gender and is crucial for valid ECG findings.  Gender-specific application of ECG electrodes can prevent misdiagnoses and unnecessary follow-up examinations.^6^ |
| **Women often present with symptoms such as abnormal fatigue, shortness of breath, and nausea.** |  correct   incorrect | Compared to men who suffer a heart attack, women often show different symptoms, particularly dyspnea and feelings of tightness or anxiety, rather than the classic retrosternal chest pressure.  Women are more likely to manifest diverse and more subtle symptoms that may not be immediately recognized as a heart attack. While men often experience classic chest pain, women are more likely to experience shortness of breath, feelings of tightness, or severe anxiety. These different presentations can lead to delayed diagnosis and treatment, as the atypical symptoms are not always recognized as signs of a heart attack. |
| **In addition to the classic risk factors, women have additional risk factors for myocardial ischemia compared to men.** |  correct   incorrect | There are several specific risk factors for Minoca that may play a role, particularly in women.   1. **Hormonal changes:** Hormonal fluctuations, especially during pregnancy and menopause, can influence the risk of Minoca. Estrogen levels can have a protective effect on the heart, and changes in these hormones could influence the risk. 2. **Pregnancy-related conditions:** Women who suffer from preeclampsia or gestational diabetes during pregnancy may have an increased risk of Minoca. 3. **Autoimmune diseases:** Women who suffer from autoimmune diseases such as rheumatoid arthritis or lupus may have a higher risk of Minoca. These diseases can affect the immune system and promote inflammation in the body. 4. **Small vessel disease:** Minoca is often associated with disorders of the small blood vessels (microangiopathy). Women may be more susceptible to microangiopathy, which increases the risk of minoca. |
| **To learn more about risk factors in pregnant women, which of the following guidelines would you use?** |  DGK Recommendations for Cardiovascular Wellness in Maternity   ESC Guidelines on the Management of Cardiovascular Diseases during Pregnancy^[[5]](#endnote-6)^   ESC Guidelines for Cardiovascular Care in Pregnancy   AWMF Recommendations for Cardiovascular Health in Pregnancy | **See, e.g. , the sections:**   - 3.3.1 Risk of cardiovascular complications in the mother; pages 3174/3175 - 4.2.1.2 Risk to the mother; page 3183 - 7.1 Etiology; page 3197 - 11.2 Risk factors for pregnancy-related venous thromboembolism and risk stratification; page 3210 |

**Important information for cardiology**

Important additional gender-specific aspects in cardiology (not taken from medical guidelines):

- **Differences in heart attack symptoms:** The typical severe chest pain that also radiates to different parts of the body occurs less frequently in women than in men. Other symptoms to look out for include shortness of breath, unusually severe fatigue, prolonged symptoms, even at rest, pain in the back and abdomen, neck or jaw, and severe, burning pressure in the chest. In women, the risk of a heart attack increases significantly with perimenopause.^[[6]](#endnote-7)^
- **Differences in blood pressure values:** 120/80 mmHg is usually used as a guideline for optimal blood pressure. However, blood pressure also varies according to gender and age. At a young age, women have lower blood pressure than men on average, but this usually rises sharply in women during perimenopause and is then slightly above the average values for men. There are no guideline-based reference values for gender and age yet. The following table shows study-based average values by age and gender.^[[7]](#endnote-8),^ ^[[8]](#endnote-9)^

| **Age** | **20-40 years** | | **40-60 years** | | **über 60 Jahre** | |
| --- | --- | --- | --- | --- | --- | --- |
|  | **women** | **men** | **women** | **men** | **women** | **man** |
| **Blood pressure (mmHg)** | 105/68 to 115/77 | 115/75 to 120/82 | 115/77 to 130/80 | 120/82 to 130/84 | 130/68 to 145/78 | 130/70 to 140/82 |
| **Source:** based on Ji et al. (2024), Figure 1^10^ | | | | | | |

1. Bundesärztekammer, Kassenärztliche Bundesvereinigung, Arbeitsgemeinschaft der Wissenschaftlichen Medizinischen Fachgesellschaften, Schaefer, C., Prien, P., Krueger, K., Ärztliches Zentrum für Qualität in der Medizin (ÄZQ), Kopp, I. & Nothacker, M. (2022). Nationale VersorgungsLeitlinie Chronische KHK - Leitlinienreport. In Ärztliches Zentrum für Qualität in der Medizin, Bundesärztekammer, Kassenärztliche Bundesvereinigung & Arbeitsgemeinschaft der Wissenschaftlichen Medizinischen Fachgesellschaften, NVL Chronische KHK. <https://doi.org/10.6101/AZQ/000490> [↑](#endnote-ref-2)
2. Ibanez, B., James, S., Agewall, S., Antunes, M. J., Bucciarelli-Ducci, C., Bueno, H., Caforio, A. L. P., Crea, F., Goudevenos, J. A., Halvorsen, S., Hindricks, G., Kastrati, A., Lenzen, M. J., Prescott, E., Roffi, M., Valgimigli, M., Varenhorst, C., Vranckx, P., Widimský, P., . . . Iakobishvili, Z. (2017). 2017 ESC Guidelines for the management of acute myocardial infarction in patients presenting with ST-segment elevation. European Heart Journal, 39(2), 119–177. <https://doi.org/10.1093/eurheartj/ehx393> [↑](#endnote-ref-3)
3. Van Gelder, I. C., Rienstra, M., Bunting, K. V., Casado-Arroyo, R., Caso, V., Crijns, H. J. G. M., De Potter, T. J. R., Dwight, J., Guasti, L., Hanke, T., Jaarsma, T., Lettino, M., Løchen, M., Lumbers, R. T., Maesen, B., Mølgaard, I., Rosano, G. M. C., Sanders, P., Schnabel, R. B., . . . Kuchkarov, H. (2024a). 2024 ESC Guidelines for the management of atrial fibrillation developed in collaboration with the European Association for Cardio-Thoracic Surgery (EACTS). *European Heart Journal*. <https://doi.org/10.1093/eurheartj/ehae176> [↑](#endnote-ref-4)
4. ESC Guideline (2020b) → doi:10.1093/eurheartj/ehaa575? [↑](#endnote-ref-5)
5. Regitz-Zagrosek, V. Roos-Hesselink, J. W., Bauersachs, J., Blomström-Lundqvist, C., Cífková, R., De Bonis, M., Iung, B., Johnson, M. R., Kintscher, U., Kranke, P., Lang, I. M., Morais, J., Pieper, P. G., Presbitero, P., Price, S., Rosano, G. M. C., Seeland, U., Simoncini, T., Swan, L., . . . Nelson-Piercy, C. (2018). 2018 ESC Guidelines for the management of cardiovascular diseases during pregnancy. European Heart Journal, 39(34), 3165–3241. <https://doi.org/10.1093/eurheartj/ehy340> [↑](#endnote-ref-6)
6. Seeland, U. (2023). Geschlechtersensible medizinische Ansätze in der Kardiologie. DMW - Deutsche Medizinische Wochenschrift, 148(09), 538–546. <https://doi.org/10.1055/a-1892-4687> [↑](#endnote-ref-7)
7. Baessler, A., Bauer, P., Becker, M., Berrisch-Rahmel, S., Goldmann, B., Grünig, E., Hamm, C., Meder, B., Kindermann, I., Ong, P., Seeland, U., Sievers, B., Strack, C., Zylla, M. M. & Boer, J. (2024b). Sex-specific aspects of cardiovascular diseases. Deleted Journal, 18(4), 293–321. <https://doi.org/10.1007/s12181-024-00694-9> [↑](#endnote-ref-8)
8. Ji, H., Kim, A., Ebinger, J. E., Niiranen, T. J., Claggett, B. L., Merz, C. N. B. & Cheng, S. (2020c). Sex Differences in Blood Pressure Trajectories Over the Life Course. JAMA Cardiology, 5(3), 255. <https://doi.org/10.1001/jamacardio.2019.5306> [↑](#endnote-ref-9)
